# Precision citrus segmentation and stem picking point localization using improved YOLOv8n-seg algorithm

**DOI:** 10.3389/fpls.2025.1655093

**Published:** 2025-09-11

**Authors:** Han Li, Zirui Yin, Zhijiang Zuo, Libo Pan, Junfeng Zhang

**Affiliations:** ^1^ State Key Laboratory of Precision Blasting, Jianghan University, Wuhan, China; ^2^ Hubei Key Laboratory of Blasting Engineering, Jianghan University, Wuhan, China; ^3^ Institute of Agricultural Mechanization, Wuhan Academy of Agricultural Sciences, Wuhan, China

**Keywords:** citrus, YOLOv8n-seg, picking point localization, instance segmentation, picking robot

## Abstract

**Introduction:**

Due to the small size of citrus stems, their color similarity to the background, and their variable position relative to the fruit, accurately locating picking points using robots in natural environments presents significant challenges.

**Methods:**

To address this issue, this study proposes a method for segmenting citrus fruits and stems based on an improved YOLOv8n-seg model, combined with geometric constraints for stem matching to achieve accurate localization of picking points. First, all standard convolutions in the model are replaced with GhostConv to reduce the number of model parameters. Furthermore, a convolutional block attention module (CBAM) and a small-object detection layer are introduced to enhance the model’s feature representation and segmentation accuracy for small objects. Then, by incorporating the positional relationship between the fruit and the stem, constraints are defined to match the target stem, and an algorithm is designed to determine the optimal picking point.

**Results:**

Experimental results show that the improved YOLOv8n-seg model achieves recall rates of 90.91% for fruits and stems, a mean average precision (mAP50) of 94.43%, and an F1-score of 93.51%. The precision rates for fruit and stem segmentation are 96.04% and 97.12%, respectively. The average detection rate of picking points reaches 88.38%, with an average localization time of 373.25 milliseconds under GPU support, demonstrating high real-time performance. Compared with other models, the improved YOLOv8n-seg model shows significantly better performance.

**Discussion:**

This study confirms the reliability and effectiveness of the proposed citrus picking point localization method and lays a technical foundation for the automated harvesting of citrus fruits.

## 1 Introduction

Citrus is an important agricultural crop in China, widely favored by consumers and holding a significant position in international markets. Since 2007, China has ranked first globally in both the planting area and yield of citrus fruits (Nie, 2023; Li et al., 2025). With increasing market demand, the scale of citrus cultivation continues to expand. However, citrus harvesting is still predominantly manual (Sun et al., 2023), which presents issues such as high labor intensity, low efficiency, and high costs (Xiao et al., 2024; Choi et al., 2025; Zhang et al., 2025). In the context of an aging population and a growing shortage of agricultural labor in China, harvesting has become the most time-consuming, labor-intensive, and costly stage in fruit production (Yin et al., 2023), severely restricting industrial development and farmer income. Therefore, promoting the mechanization and intelligent transformation of citrus harvesting has become essential, and the development of harvesting robots is a vital approach to addressing this issue.

In the complex natural environment of orchards, the key to achieving efficient and non-destructive picking by harvesting robots lies in the accurate recognition of fruits and their picking points (Hou et al., 2024; Liang et al., 2025). Currently, visual recognition in harvesting robots is mainly realized through traditional image processing methods and deep learning algorithms (Fu et al., 2024). Traditional image processing relies on manually extracted image features for object recognition. (Xu and Lü, 2015). extracted color features of bayberry images and used the Hough transform to fit fruit contours for recognition. However, this method requires high-quality images and can only identify a small number of fruits. Although traditional methods have shown some success in fruit recognition, they lack robustness and adaptability due to limited feature extraction and susceptibility to changes in lighting, background, and fruit color (Xiao et al., 2023; Liu et al., 2024; Li et al., 2025). With the rapid development of machine vision, deep learning techniques have been widely applied to fruit detection tasks due to their high accuracy and efficiency (Li et al., 2025; Zhao et al., 2025), such as blueberries (Wang et al., 2024), apples (Zhang and Zhang, 2024), bananas (Wu et al., 2024), pears (Ren et al., 2025), and tomatoes (Huang et al., 2025). Among these, the single-stage object detection algorithm YOLO (Redmon et al., 2015) has attracted significant attention in the field. This algorithm can directly input standardized images into a convolutional neural network for object detection. (Xu et al., 2023). proposed a high-precision lightweight detection method based on YOLOv4, using the lightweight feature extraction network GhostNet to enhance citrus feature representation, achieving an accuracy of 93.45%. (Liu et al., 2024). developed a lightweight algorithm, Faster-YOLO-AP, based on YOLOv8n-seg, which achieved efficient and accurate apple detection. However, these studies mainly focus on fruit detection, with picking points typically located on the fruit itself, which is suitable for harvesting fruits with hard skins. For citrus fruits with thin peels and soft flesh, picking directly from the fruit can easily cause mechanical damage. Therefore, the picking point should be positioned on the fruit stem to ensure the integrity of the fruit during harvesting. Compared to fruit recognition, stem detection presents greater challenges due to the small size of the stem, its color similarity to stems and leaves, and its low pixel ratio in the image, which categorizes it as a typical small object. Against this background, segmentation methods based on deep learning, which offer more precise contour extraction, have become a focal point for stem detection research. (Su et al., 2025). introduced the AP-UNet model to accurately detect guava fruits and stems at night. (Yang et al., 2023). adopted the YOLOv5s-seg segmentation model to detect strawberry stems on trellises, achieving a precision of 82.74%, recall of 82.01%, and mean average precision of 80.67%. (Baek et al., 2025). proposed the AppleStem (AS)-YOLO model, utilizing ghost bottlenecks and global attention mechanism to segment apple stems. Compared to detection, segmentation methods provide greater advantages in handling weak-feature and small-scale targets, providing more precise information for the determination of cutting locations by harvesting robots. Based on this, this study proposes an improved YOLOv8-seg model to enhance the segmentation performance for citrus stem.

Most existing stem picking point detection methods infer stem positions based on fruit locations, showing good adaptability for crops like kiwifruit, lychee, and apples, where stems are generally located directly above the fruit (Li et al., 2022). (Li et al., 2025). determined stem picking points for kiwifruit by searching for fruit pixel features along the stem’s vertical extension and establishing a subordinate relationship between fruit and stem. (Xie et al., 2024). proposed a compound model-based visual localization method, using object detection and instance segmentation to continuously detect strawberries and segment their stems, identifying picking points and stem tilt angles. (Zhang et al., 2024). optimized the YOLOv5s model to detect mango stems via segmentation for cluster harvesting. These studies effectively realized stem detection and picking point localization based on the physical connection between fruit and stem. However, due to tree structure and gravity, citrus fruits often hang at inclined angles ranging from 30° to 60°, or even upside down, resulting in significant stem position variability and increased detection difficulty.

In summary, this study focuses on the dwarf-cultivated citrus variety “Dafen No. 4” and proposes a segmentation method based on an improved YOLOv8n-seg model to enhance the detection accuracy of citrus fruit and stems in natural environments. Based on this, a stem matching method using geometric constraints is designed to establish the correspondence between fruit and stem, identify the target stem, extract the skeleton line, and develop an algorithm to localize the optimal picking point. This method provides both theoretical and technical support for improving the robustness and practicality of intelligent orchard harvesting systems.

## 2 Materials and methods

### 2.1 Citrus dataset construction

#### 2.1.1 Image acquisition

All citrus images used in this study were collected from the citrus cultivation area of the Wuhan Academy of Agricultural Sciences, Hubei Province, China (latitude 30°43’30’’ N, longitude 114°30’15’’ E). The cultivated variety used for image acquisition was ‘Dafen No. 4’. Images were captured using an Intel RealSense D435i depth camera with a resolution of 1920×1080 pixels. All data were collected during daytime to avoid insufficient lighting conditions that could negatively affect image quality and subsequent visual processing. During image acquisition, the relative position between the camera and the canopy surface of the citrus trees was carefully controlled to maintain a parallel distance of 0.3 to 0.6 meters. This ensured that the captured images accurately reflected the natural growth state of the fruit. Considering the complexity of the orchard environment and the safety of the research personnel, data collection was not conducted under extreme weather conditions such as heavy rainfall or low-light at night. This approach was adopted to minimize the impact of adverse conditions factors on data quality.

**Figure 1:**
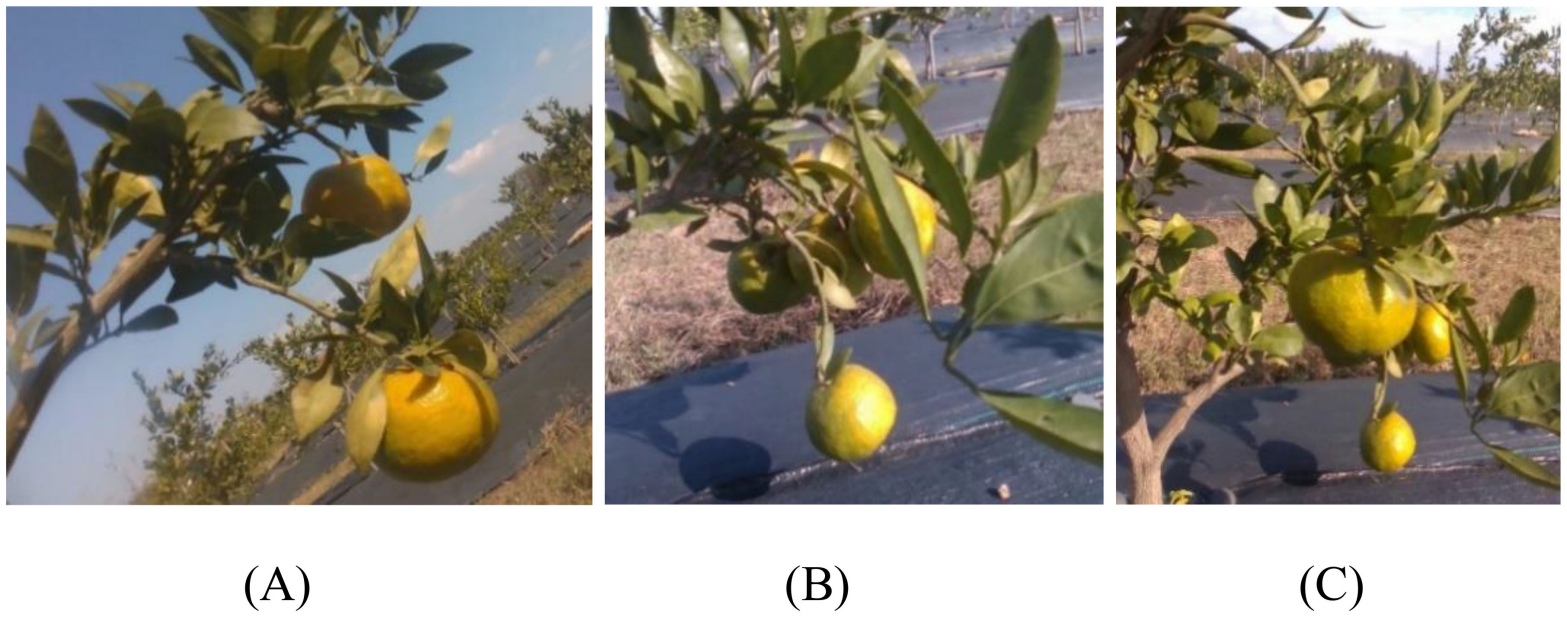
Example of a citrus image. **(A)** No occlusion **(B)** Occlusion **(C)** Sufficient illumination.

After image collection, the raw images were screened to remove blurred, duplicate, or other invalid images. A total of 1,568 clear original citrus images were retained. Representative samples are shown in **Figure 1**. To enhance the diversity of the dataset and improve the model’s robustness under complex conditions such as varying shooting angles, different levels of occlusion, and motion blur, several data augmentation techniques were applied. These included flipping, rotation, translation, brightness adjustment, noise addition, and Gaussian blurring, expanding the dataset to 4,023 images. Subsequently, a secondary screening was performed to eliminate lower-quality images, resulting in a final dataset of 4,000 high-quality citrus images.

#### 2.1.2 Image annotation

**Figure 2:**
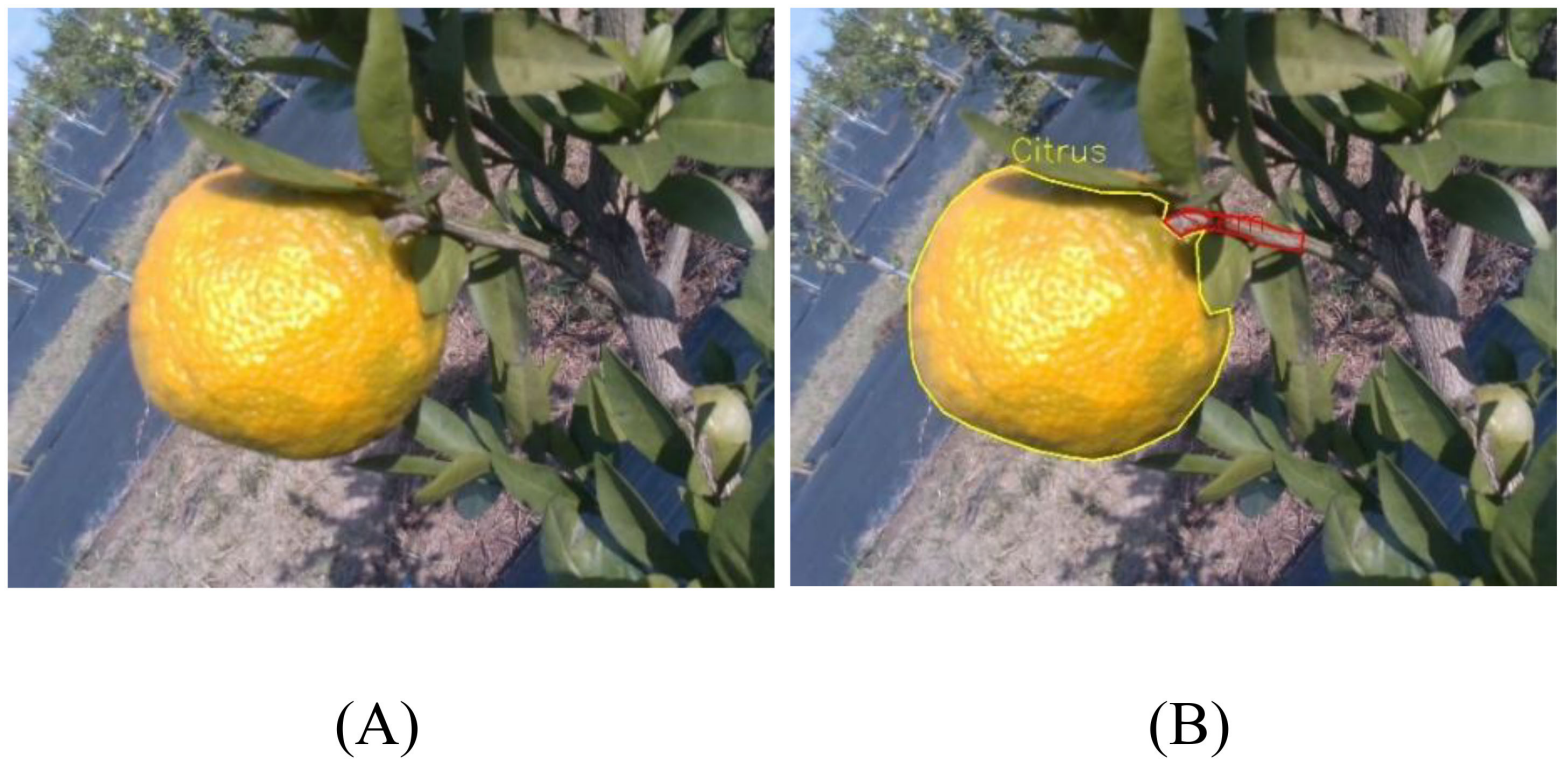
Data annotation. **(A)** Original image **(B)** Annotation visualization.

In this study, Labelme software was used to manually annotate the images, establishing two categories: Citrus Fruit (Citrus) and Citrus Stem (Stem). During annotation, fruits and stems that were severely occluded or difficult to identify due to poor lighting were not annotated. To ensure data consistency and proper correspondence, a one-to-one annotation strategy was adopted for fruits and their associated stems. As shown in **Figure 2**, annotation was performed using polygonal outlines to trace the contours of both the citrus fruits and stems. All annotation files were then converted into TXT format to be compatible with the input format required by the segmentation network model. Finally, the dataset was randomly divided into training, validation, and test sets in an 8:1:1 ratio. This ensured balanced data distribution during model training and improved the model’s generalization ability. Following the dataset partitioning, a statistical analysis of class distribution was conducted across the training, validation, and test sets. The results indicate that the proportions of citrus fruits and stems are comparable across the three subsets, reflecting a well-balanced and consistent data distribution. **Table 1** presents the distribution of the citrus image dataset.

**Table 1:** Distribution of citrus image datasets.

Dataset type	Number of images/pieces	Number of citrus fruits/pieces	Number of citrus stems/pieces
Training set	3200	5330	4645
Verification set	400	650	550
Test set	400	655	560
Total	4000	6635	5755

Dafen No. 4 is a newly emerging citrus variety with high-quality peel and pulp characteristics. To enable non-destructive harvesting without damaging the peel or injuring the pulp, the picking robot is required to perform the cutting operation on the stem at a certain distance from the fruit. As the stems of this variety are generally long, annotating the entire stem would not only increase the complexity of data annotation but also introduce redundant information. Therefore, in this study, image annotation focuses only on the area near the connection between the fruit and the stem. The central part of the segmented stem mask is selected as the picking point region. This strategy effectively captures the relevant features of the picking point while simplifying the annotation task, providing valuable data support for model training and picking point localization.

### 2.2 Citrus fruit and stem segmentation network

#### 2.2.1 YOLOv8-seg segmentation model

YOLOv8-seg is an advanced instance segmentation model developed by the Ultralytics team as part of the YOLO series. It inherits the efficiency and speed of its predecessors, while incorporating several architectural optimizations. The overall architecture consists of four components: the Input layer, Backbone, Neck, and Head. Compared to the earlier YOLOv5-seg model, YOLOv8-seg replaces the original C3 module with a C2f module, enhancing feature reuse and gradient flow, thereby improving feature representation. In addition, it removes convolution operations in the upsampling stage and employs a decoupled detection head, which separates classification and bounding box regression tasks to improve detection performance. YOLOv8-seg also supports pixel-level multi-object segmentation, making it suitable for tasks that demand precise object contours. The primary reason for selecting YOLOv8-seg as the baseline model in this study, rather than adopting a newer version, lies in its relatively stable architecture, well-established performance, and extensive validation. These characteristics provide a reliable foundation for subsequent network structure optimization and functional module integration.

#### 2.2.2 Improved YOLOv8n-seg segmentation model

**Figure 3:**
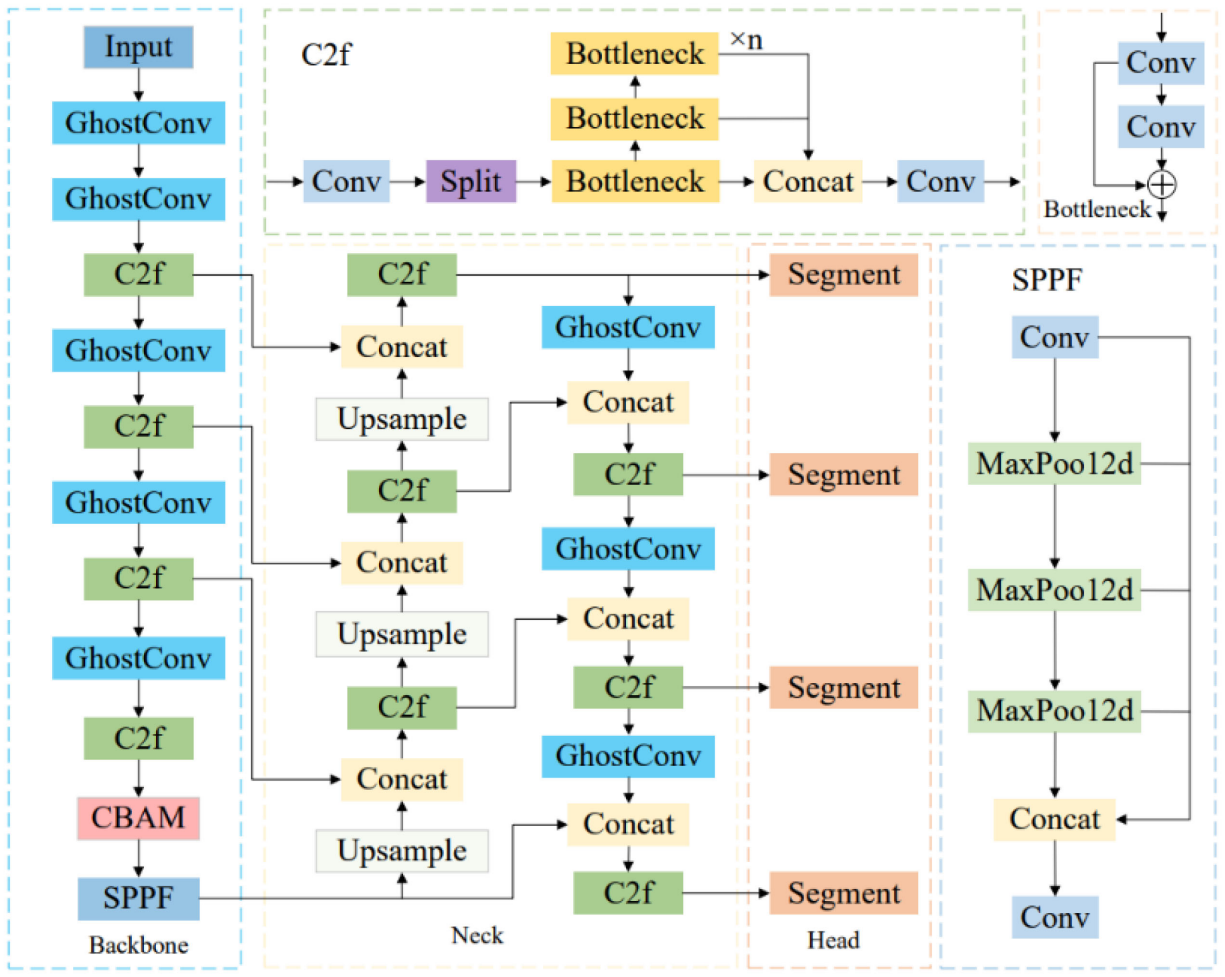
Architecture of the improved YOLOv8n-seg model.

YOLOv8n-seg, as the lightest segmentation model in the YOLOv8 series, offers fast inference speed and high detection accuracy. However, in practical orchard environments, the segmentation of citrus fruits and stems still faces numerous challenges. The small size of stems, their color similarity to the background, frequent occlusions, and varying lighting conditions all increase the difficulty of accurate target segmentation. To address these issues, this study proposes targeted structural improvements to the YOLOv8n-seg model based on the specific perception requirements of citrus harvesting tasks, aiming to enhance the model’s ability to segment critical regions. First, the GhostConv module is introduced to replace the original standard convolution, reducing the number of model parameters and achieving a lightweight network structure suitable for resource-constrained agricultural scenarios. Second, the CBAM attention mechanism is embedded into the backbone to strengthen the model’s responsiveness to key features such as stems, thereby improving segmentation accuracy under complex backgrounds or partial occlusion conditions. Finally, a small-object detection layer is added to enhance the model’s multi-scale perception capability, further improving the detection of small stem targets and thereby increasing the completeness and robustness of picking point localization. The improved YOLOv8n-seg network architecture is illustrated in **Figure 3**.

(1) Lightweight network reconstruction.

Traditional convolution operations typically stack multiple convolutional kernels and apply them to all channels of the input feature map. While this approach allows for the extraction of rich image features, it results in slow computation and a large number of parameters, which limits the deployment efficiency of models on resource-constrained devices. To alleviate this burden, previous studies have proposed lightweight convolutional networks such as ShuffleNet and MobileNet by optimizing convolutional structures for more efficient computation (Howard et al., 2017; Zhang et al., 2017). However, traditional convolution operations still consume significant memory resources. To address this issue, the present study introduces the GhostConv module (Han et al., 2020) as a replacement for standard convolution operations, aiming to improve feature representation efficiency and reduce the overall complexity of the network.

**Figure 4:**
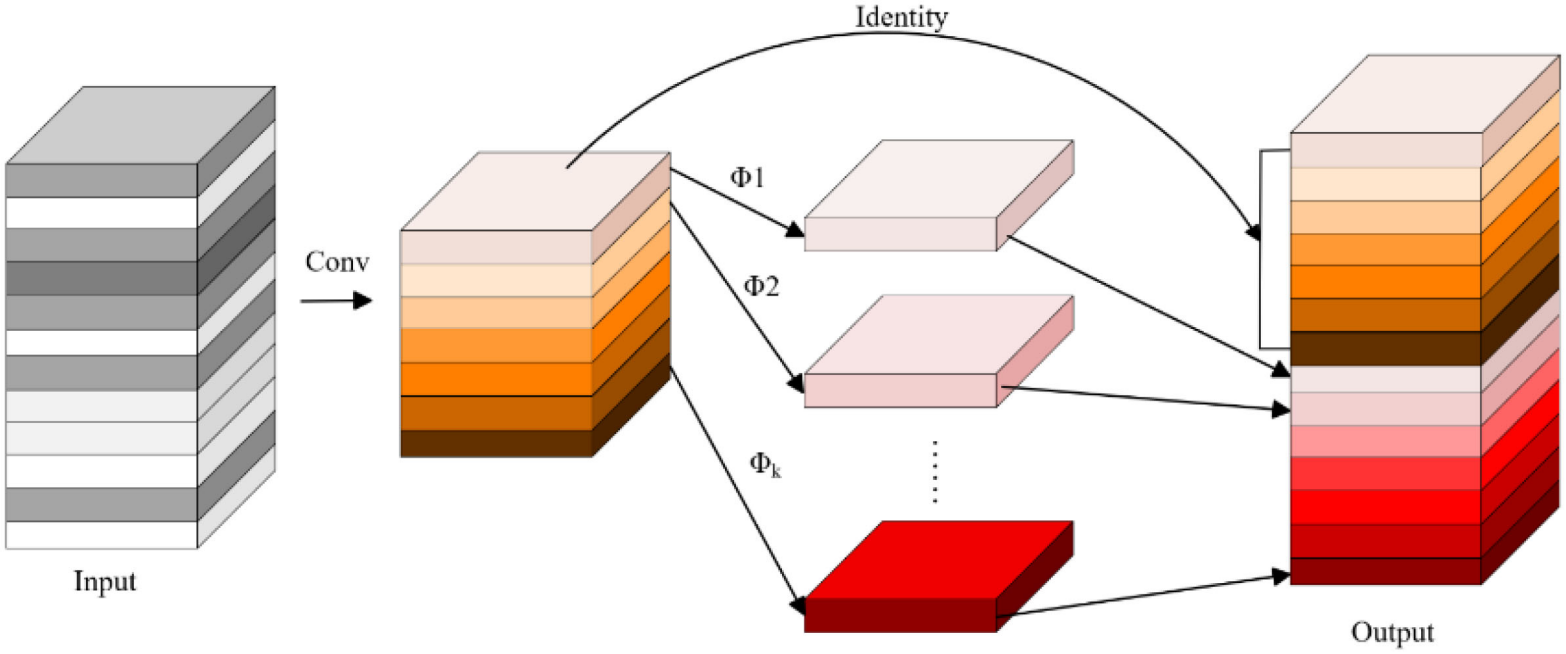
Structure of the GhostConv module.

As shown in **Figure 4**, the GhostConv module mainly consists of three main steps. First, some feature maps are generated using standard convolution operations. Then, a series of linear transformations are applied to these feature maps to produce additional feature maps. Finally, both sets of feature maps are concatenated to form the complete output. This structure effectively reduces the computational cost associated with learning non-critical image features, thereby improving the feature representation efficiency of the network while maintaining model accuracy and reducing parameter count.

(2) Incorporation of attention mechanism.

**Figure 5:**
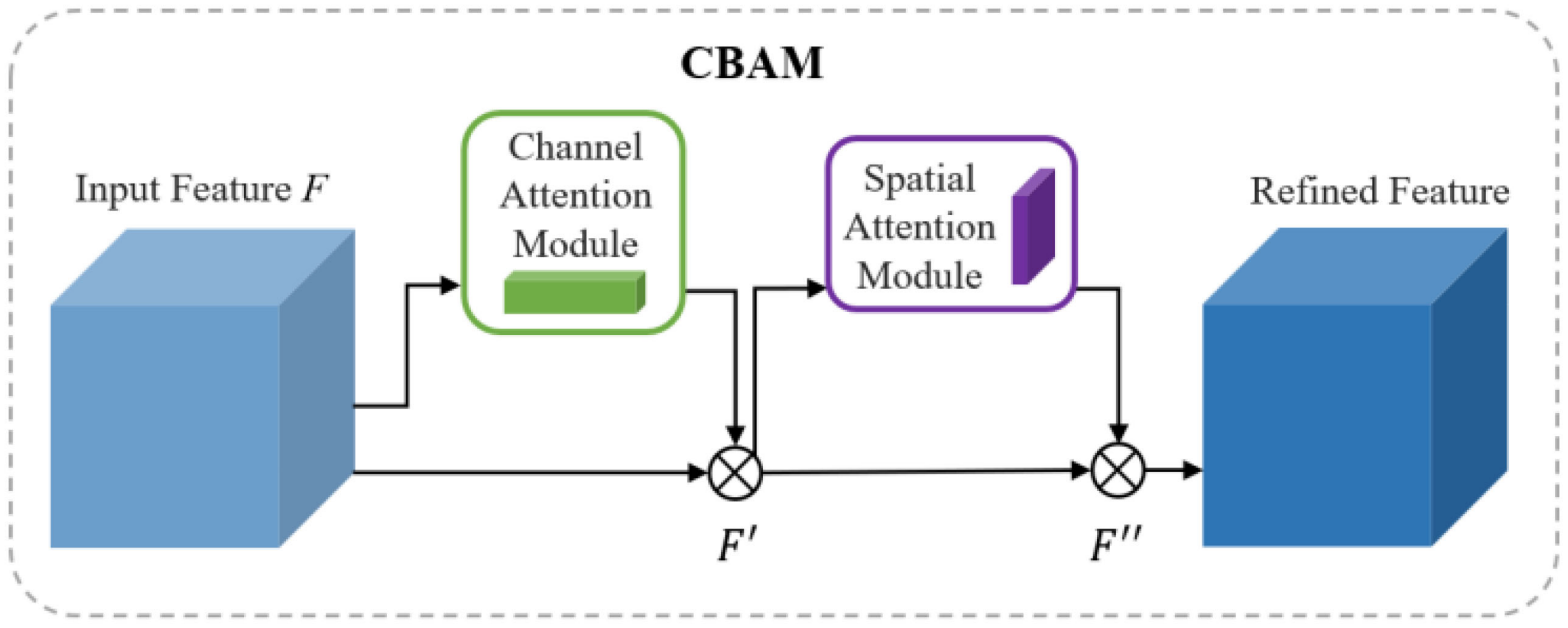
Architecture of the CBAM.


In the complex environment of orchards, factors such as varying lighting conditions, occlusion by branches and leaves, and unstructured backgrounds introduce significant interference, resulting in a large amount of irrelevant information in the images. These challenges severely affect the model’s ability to accurately recognize target objects. Attention mechanisms, which assign adaptive weights to feature maps, enable the model to dynamically select and enhance key information. This can effectively improve the model’s discriminative capacity under complex background conditions. Therefore, in this study, the CBAM (Woo et al., 2018) is integrated into the backbone of the YOLOv8n-seg model to enhance the network’s ability to represent target regions. The CBAM module is composed of a channel attention module (CAM) followed by a spatial attention module (SAM). CAM captures the importance of different channels by applying both global average pooling and max pooling, emphasizing features related to fruits and stems. SAM models the spatial attention distribution using the feature map compressed along the channel dimension, improving the model’s response to the edges of small targets under complex backgrounds. While maintaining a lightweight architecture, the CBAM module enhances the network’s perception of target regions. **Figure 5** shows the structural diagram of the CBAM module. Given an input feature map *F∈R^C×H×W^*, CBAM sequentially derives the CAM map *M_C_∈R^C×1×1^* and the SAM map *M_S_∈R^1×H×W^*. The overall process is as follows:

$$F'=M_{C}\left(F\right)⊗F$$

$$F''=M_{S}\left(F'\right)⊗F'$$

Here, ⊗ denotes element-wise multiplication. *F’* represents the intermediate feature map after channel attention adjustment, and *F”* is the final enhanced output feature map.

(3) Introduction of small object detection layer.

To enhance the model’s ability to detect and segment small-scale targets such as citrus stems, this study introduces an additional shallow detection path on top of the original three-scale segmentation structure (P3, P4, P5) of YOLOv8n-seg. This new path constructs a fourth output branch by incorporating upsampling and feature fusion operations on shallow feature maps, aiming to compensate for the original segmentation head’s limited perception of small targets. Specifically, this path leverages the rich spatial structure information in low-level feature maps and enhances it by fusing semantic features from deeper layers, thereby improving the model’s perception consistency across different object scales. Considering that citrus stems are small, slender, and prone to occlusion by branches and leaves, this architectural design enhances the model’s representational capacity for such targets, resulting in more stable segmentation performance under complex backgrounds. Moreover, the added module maintains the lightweight nature of the overall network, facilitating deployment on resource-constrained intelligent agricultural machinery with high practical applicability.

### 2.3 Citrus stem picking point localization method

#### 2.3.1 Target stem determination strategy

**Figure 6:**
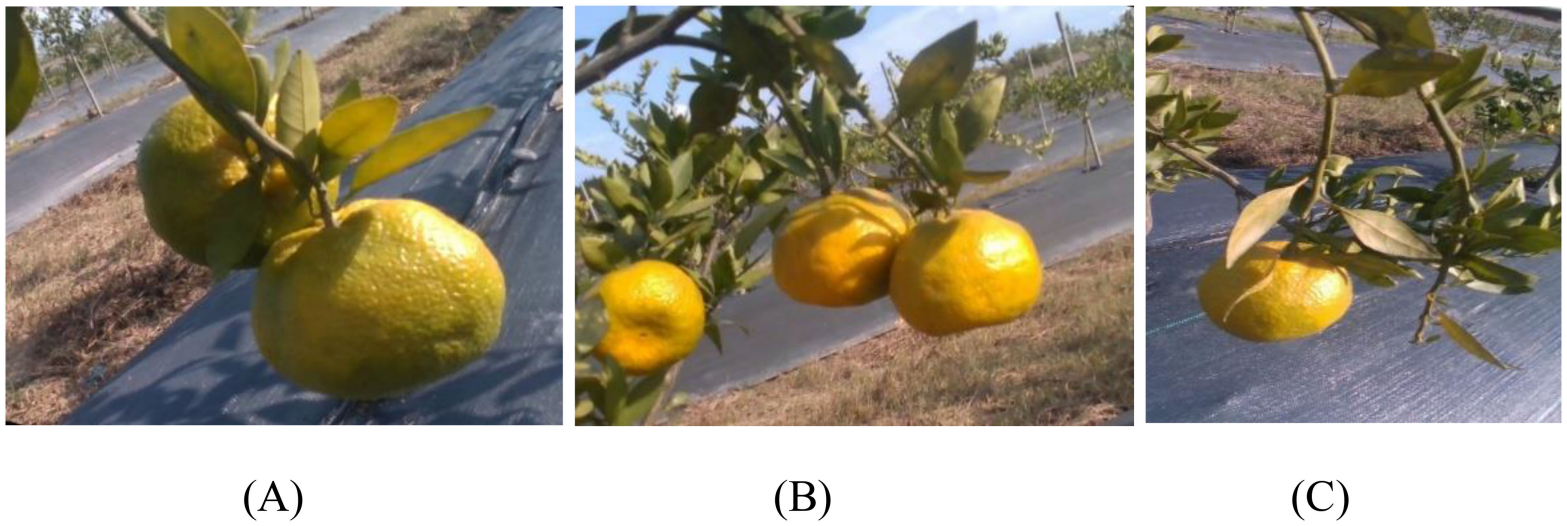
Relationship between citrus fruits and their stems. **(A)** One stem corresponds to two fruits; **(B)** fruits are clustered with stem occlusion; **(C)** no fruit below the stem.

As described in Section 2.2.2, after the citrus fruit and stem are segmented, it is necessary to further determine whether the detected stem region is the target stem connected to the corresponding fruit in order to accurately identify the picking position. However, as shown in **Figure 6**, complex situations commonly occur in the tree canopy, including multiple fruits on a single stem, fruit clusters, occlusion by stems, and fruitless stems. These factors make it difficult to directly establish the correspondence between fruits and stems. To address this challenge, this study proposes a stem matching method based on geometric constraints. First, the segmented stem region is fitted with a straight line using the least squares method to estimate its general geometric direction. Then, the Euclidean distance from each fruit centroid to the fitted line is calculated, and the stem with the shortest distance is selected as the preliminary candidate. Finally, by comparing the maximum distance from the centroid to the fruit contour with the minimum distance from the centroid to the fitted stem line, the final target stem is identified.

First, linear fitting is performed on the segmented stem regions to extract their geometric directional features. The least squares method is used to fit a straight line to the contour points of each stem. Suppose there are N stems in the image, forming a set $B={\left\{l_{i}\right\}}_{i=1}^{N}$ . Each stem *l*_*i*_ is represented by a fitted line with slope *a*_*i*_ and intercept *b*_*i*_. The equation of the fitted line for the i-th stem is expressed as: *y*=*a*_i_ *x*+*b*_i_.

Next, to accurately obtain the centroid coordinates of the citrus fruits, it is necessary to parse the label files generated by the improved YOLOv8n-seg model and extract the fruit segmentation masks. Since fruits may be clustered or touching, directly calculating the centroid could lead to miscounting the number of fruits. Within the detected citrus region R, which contains M pixels, the centroid coordinates (*x*_*c*_, *y*_*c*_) are calculated as follows:


$$x_{c}=\frac{1}{M}\sum_{\left(x,y\right)\in R}x,y_{c}=\frac{1}{M}\sum_{\left(x,y\right)\in R}y$$

To establish the correspondence between fruits and stems, it is necessary to calculate the distance from the fruit centroid (*x*_*c*_, *y*_*c*_) to all fitted stem lines. The calculation formula is as follows:


$$d_{i}=\frac{\left|a_{i}x_{c}-y_{c}+b_{i}\right|}{\sqrt{a_{i}^{2}+1}}$$

Obviously, the smaller the distance *d*_*i*_, the more likely the stem *l*_*i*_ corresponds to the target stem of the fruit. Let the minimum distance be denoted as *d*_1_. Then, the candidate target stem should satisfy the following equation, and the corresponding stem *l*_min_ is selected as the candidate target stem:


$$l_{min}=\arg min_{l_{i}\in B}d_{i}$$

Let the set of boundary points on the fruit’s maximum contour be $D={\left\{\left(x_{j},y_{j}\right)\right\}}_{j=1}^{M}$ .Then, the shortest distance from the centroid to the boundary is defined as:


$$d=min_{\left(x_{j},y_{j}\right)\in D}\sqrt{{\left(x_{C}-x_{j}\right)}^{2}+{\left(y_{C}-y_{j}\right)}^{2}}$$

**Figure 7:**
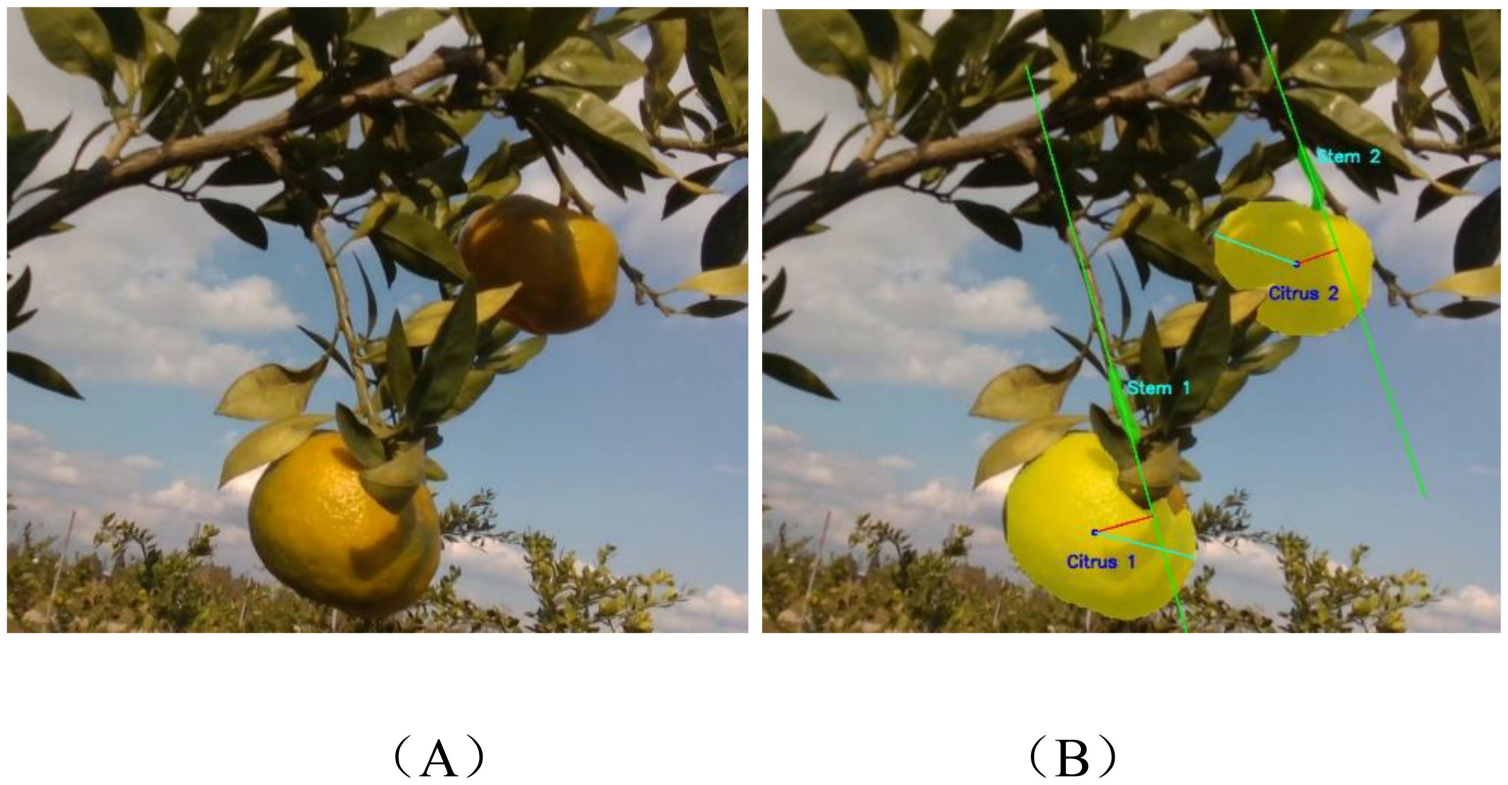
Visualization of citrus fruit–stem matching: **(A)** Original image; **(B)** Matching result image.

Since the boundary points of the fruit contour represent its maximum physical extent, the maximum distance *d* from the fruit centroid to the contour boundary can be used as a constraint for matching. This ensures that the matched stem is structurally connected to the corresponding fruit. The constraint helps eliminate stems that are not clearly associated with any fruit, thereby improving the accuracy of the matching process. If the shortest distance *d*_1_ from the fruit centroid to the fitted stem line satisfies the condition *d*_1_< *d*, the stem is considered to be validly matched with the fruit and identified as the target stem. Otherwise, it is regarded as unmatched. A visual example of citrus fruit and stem matching is shown in **Figure 7**. In **Figure 7B**, the green lines (Stem 1 and Stem 2) represent the stem orientations estimated using the least squares fitting method. The red lines indicate the shortest distance from the centroid of each fruit to its corresponding stem direction, while the cyan lines represent the maximum distance from the fruit centroid to its contour boundary. Based on these geometric constraints, the structural association between each fruit and stem can be determined. The figure illustrates the matching relationships, such as “Citrus 1 - Stem 1” and “Citrus 2 - Stem 2,” providing an intuitive visualization of the fruit-to-stem matching process.

#### 2.3.2 Stem picking point localization

**Figure 8:**
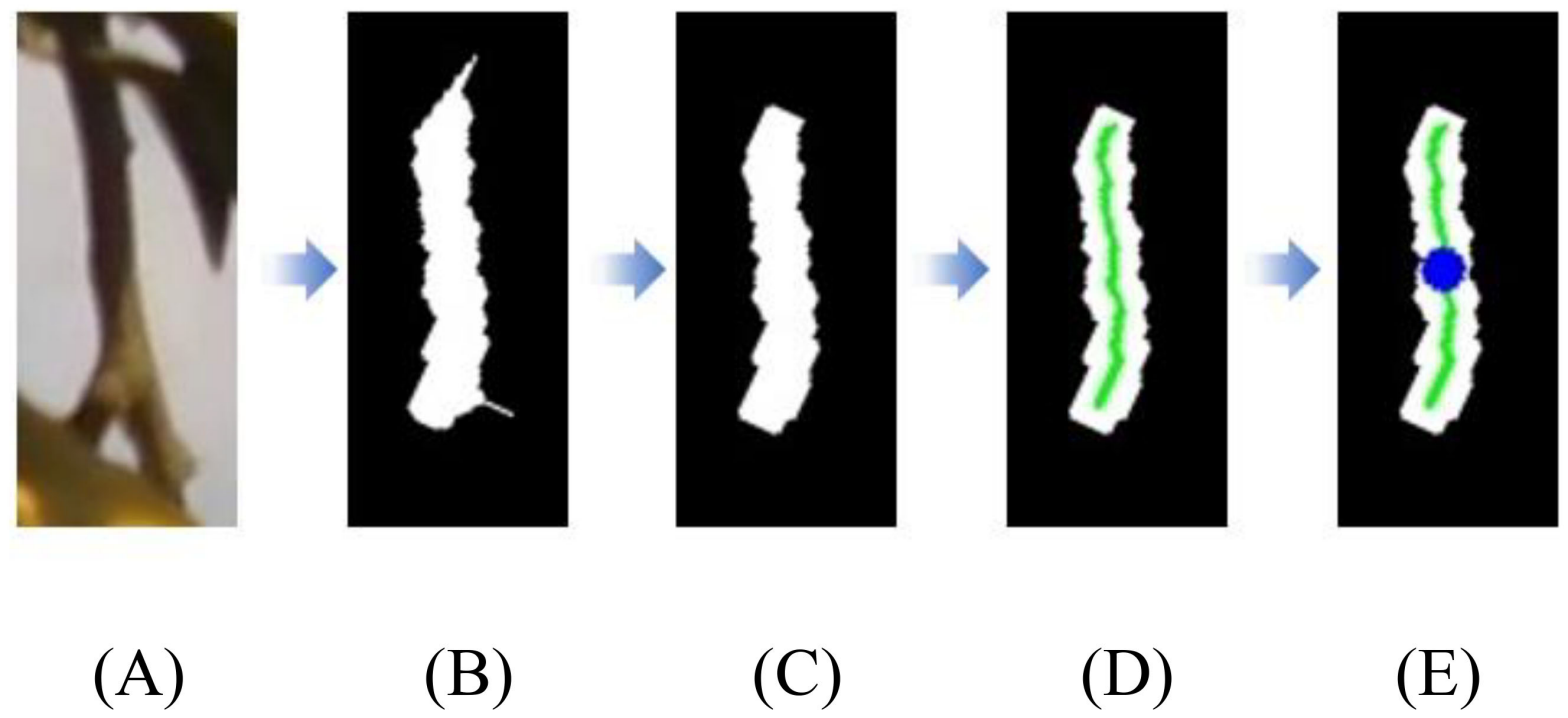
Flowchart of the picking point localization strategy. **(A)** Stem region of interest; **(B)** Image binarization; **(C)** Morphological processing; **(D)** Skeleton extraction; **(E)** Picking point localization.

After matching the target stem using the geometric constraint method, further localization of the picking point on the target stem is necessary to achieve automated harvesting. First, the stem segmentation mask output by the YOLOv8n-seg model is used to extract the region of interest (ROI), which is then converted into a corresponding binary image. This image is then processed using morphological opening operations to eliminate falsely connected regions and edge noise, thereby enhancing the main structure of the stem. Next, an image thinning algorithm is applied to extract the skeleton of the preprocessed image, allowing for simplification of the stem’s structural information by identifying its central axis. Based on this skeleton, the midpoint of the centerline is selected as the picking point, and its spatial coordinates are obtained through coordinate transformation. The flowchart of the picking point localization strategy is shown in **Figure 8**.

## 3 Experimental results

### 3.1 Experimental environment and parameter configuration

The model training and testing in this study were conducted under the same environment. The host operating system was Windows 11, with a Gen Intel(R) Core(TM) i7-12650H CPU @ 2.3GHz, 32 GB RAM, and an NVIDIA GeForce RTX 4060 Laptop GPU. The neural network was trained in an Anaconda 3 virtual environment using the PyTorch 2.5.1 deep learning framework, configured with Python 3.8, and supported by CUDA 11.8 and cuDNN 9.1.0 for GPU parallel computing. Detailed training parameters of the model are listed in **Table 2**.

**Table 2:** Model training parameters.

Parameter category	Parameter setting
Initial learning rate	0.01
Number of iteration rounds	200
Batch size	8
Picture size	640×640
Optimizer	Stochastic gradient descent(SGD)
Momentum parameter	0.937

### 3.2 Segmentation evaluation metrics

In this study, the model performance was evaluated using several metrics, including Precision (P), Recall (R), mean Average Precision at IoU threshold 0.5 (mAP0.5), F1-score, number of parameters, and model size. Precision measures the accuracy of the segmentation results, representing the proportion of correctly predicted target pixels among all pixels predicted as target regions. Recall indicates the completeness of the segmentation, referring to the proportion of correctly identified target pixels among all actual target pixels. Average Precision is the mean of precision values at different recall levels. The mAP0.5 represents the average AP across all classes when the Intersection over Union (IoU) threshold is set to 0.5, and it is used to comprehensively assess the overall performance of the model in citrus fruit and stem segmentation tasks. The F1-score is the harmonic mean of precision and recall, reflecting the model’s balanced performance in terms of accuracy and completeness. The corresponding formulas are defined as follows:


$$Precision=\frac{TP}{TP+FP}$$

$$\text{Recall}=\frac{TP}{TP+FN}$$

$$AP=\int_{0}^{1}P\left(R\right)dR$$

$$mAP=\frac{1}{N}\sum_{i=1}^{N}AP_{i}$$

$$\text{F}1=\frac{2PR}{P+R}$$

In the above formula, TP is the count of correctly predicted positives; FP is the count of negatives wrongly predicted as positives; FN is the count of positives wrongly predicted as negatives; N is the number of classes, which is 2 in this study.

### 3.3 Segmentation evaluation metrics

**Figure 9:**
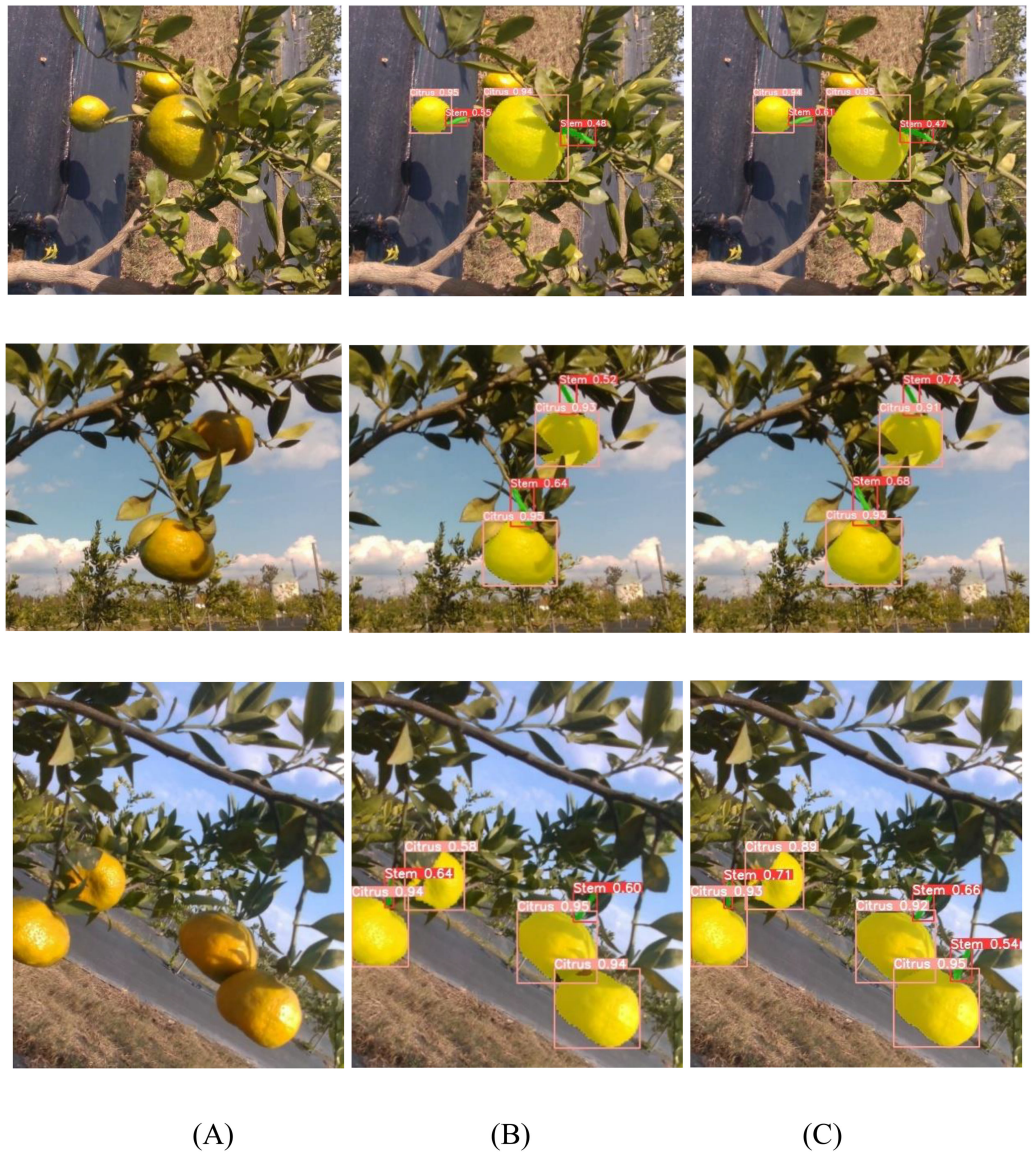
Comparison of segmentation results. **(A)** Original image; **(B)** Segmentation result using the original YOLOv8n-seg model; **(C)** Segmentation result using the improved YOLOv8n-seg model.

To visually demonstrate the performance of the model in citrus image segmentation, three representative images were randomly selected from the test set for comparative analysis, as shown in **Figure 9**. It can be observed that the improved YOLOv8n-seg model outperforms the original model in both object detection and segmentation performance. Specifically, the enhanced model is capable of identifying target regions missed by the original model, resulting in more complete segmentation outputs. In addition, the improved model exhibits generally higher confidence scores in the segmentation results, demonstrating stronger discriminative capability and localization accuracy, thereby enhancing the reliability and practical applicability of the segmentation outcomes.

### 3.4 Ablation study

To verify the impact of each module on model performance, ablation experiments were conducted on the small object detection layer, the lightweight GhostConv module, and the CBAM attention mechanism. The results are shown in **Table 3**. The baseline model without any improvements achieved an overall precision of 93.08%. The segmentation precision for fruits and stems was 96.25% and 89.91%, respectively. The recall reached 88.83%, mAP50 was 91.71%, and the F1-score was 90.81%.

**Table 3:** Results of the ablation study.

Small target detection layer	GhostConv	CBAM	P/%	P_Fruit_/%	P_Stem_/%	R/%	mAP50/%	F1/%	Parameters/M	Model size/MB
—	—	—	93.08	96.25	89.91	88.83	91.71	90.81	3.26	6.45
✓	—	—	95.36	96.51	94.21	90.35	93.71	92.64	3.24	6.53
✓	✓	—	96.13	95.99	96.27	90.06	93.45	92.84	2.93	5.96
✓	✓	✓	96.56	96.04	97.12	90.91	94.43	93.51	2.94	5.98

After adding the small object detection layer, all performance metrics improved. Precision, recall, mAP50, and F1-score increased to 95.36%, 90.35%, 93.71%, and 92.64%, respectively. The stem segmentation precision rose to 94.21%. Although the model size slightly increased, the parameter count changed marginally, and the performance gain was significant, indicating the module’s effectiveness in enhancing small object segmentation. Subsequently, replacing standard convolution with the GhostConv module reduced the number of parameters from 3.24M to 2.93M and the model size from 6.53MB to 5.96MB, achieving a more lightweight architecture. While maintaining high accuracy, recall and F1-score improved slightly. The segmentation precision for fruits and stems reached 95.99% and 96.27%, respectively, demonstrating a good balance between performance and efficiency. Finally, incorporating the CBAM attention mechanism further enhanced the model. The precision, recall, mAP50, and F1-score increased to 96.56%, 90.91%, 94.43%, and 93.51%, respectively. Fruit segmentation precision remained at 96.04%, while stem segmentation precision increased to 97.12%. Despite a slight increase in parameter count and model size, the model remained lightweight and highly deployable.

### 3.5 Comparative experiments of different algorithms

To validate the performance of the improved YOLOv8n-seg model in citrus fruit and stem segmentation tasks, we conducted comparative experiments against YOLOv5n-seg, YOLOv6n-seg, YOLOv9s-seg, and the original lightweight YOLOv8n-seg model. The results are shown in **Table 4**.

**Table 4:** Comparative results of different models.

Model	P/%	R/%	mAP50/%	F1/%	Parameters/M	Model size/MB
YOLOv5n-seg	91.31	86.17	89.07	88.51	2.76	5.51
YOLOv6n-seg	94.59	88.99	91.76	91.57	4.40	8.62
YOLOv9s-seg	94.01	90.67	93.41	92.24	7.55	15.20
YOLOv8n-seg	93.08	88.83	91.71	90.81	3.26	6.45
Improve YOLOv8n-seg	96.56	90.91	94.43	93.51	2.94	5.98

The improved YOLOv8n-seg model demonstrated superior performance across key metrics in the segmentation task. The model outperformed the baseline models in terms of precision, recall, mAP0.5, and F1-score, reaching 96.56 percent, 90.91 percent, 94.43 percent, and 93.51 percent, respectively. Compared with the original YOLOv8n-seg model, these metrics increased by 3.48 percent, 2.08 percent, 2.72 percent, and 2.70 percent, respectively. Furthermore, in comparison with the YOLOv5n-seg, YOLOv6n-seg, and YOLOv9s-seg models, the mAP0.5 of the improved model increased by 5.36 percent, 2.67 percent, and 1.02 percent, respectively, indicating enhanced segmentation performance. In terms of model complexity, the improved YOLOv8n-seg achieved a parameter count of 2.94 million and a model size of 5.98 megabytes, maintaining a lightweight architecture while significantly improving performance. Compared with YOLOv5n-seg, the model showed a slight increase in parameter size but delivered substantial performance gains. Compared with the more complex YOLOv9s-seg, the improved model achieved higher segmentation accuracy with reduced complexity. The model achieves favorable segmentation performance for citrus fruits and stems, while maintaining a smaller model size and higher detection accuracy.

### 3.6 Citrus picking point localization experiments

To evaluate the adaptability and accuracy of the citrus picking point localization method in complex real-world orchard environments, RGB images captured under natural orchard conditions using a D435i depth camera were selected for localization experiments. A total of 100 images were randomly sampled from the test dataset, comprising 155 annotated picking points and covering diverse conditions including varying lighting, viewing angles, and backgrounds. Images were categorized into three scenarios based on the visibility of fruits and stems: no occlusion, mild occlusion, and moderate occlusion. Mild occlusion indicates that both fruits and stems have over 60% visible area, while moderate occlusion refers to visibility between 30% and 60%. The citrus picking point localization results under different scenarios are presented in **Table 5**. The improved YOLOv8n-seg model correctly segmented 150 target stems, with 137 picking points accurately localized. Five localization errors were caused by stem segmentation failures, and 13 failures were due to stem occlusion, resulting in an average picking point detection rate of 88.38%. Additionally, with GPU support, the system’s average processing time from input image to completion of both segmentation and picking point localization was 373.25 milliseconds, demonstrating strong real-time performance and practical deployment potential.

**Table 5:** Experimental results of citrus picking point localization under different scenarios.

Scene type	Image count	Total targets	Accurate picking point localization	Segmentation failures	Stem occlusion failures	Average localization time (ms)
No obstruction	30	32	29	1	2	328.33
Mild occlusion	30	46	41	1	4	422.67
Moderate occlusion	40	77	67	3	7	368.75
Total/Average	100	155	137	5	13	373.25

**Figure 10:**
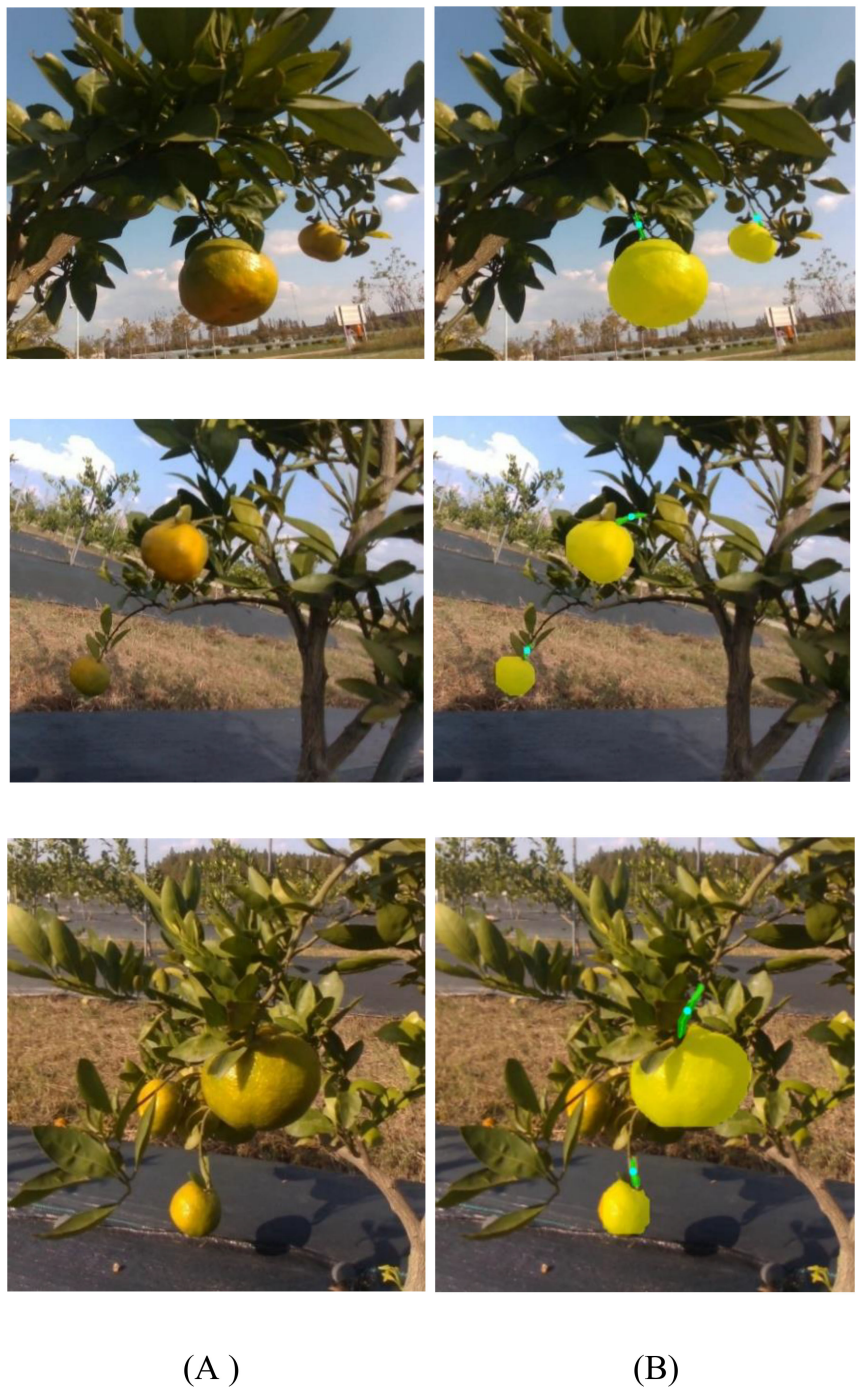
Visualization of citrus picking point localization in orchard environments. **(A)** Original images; **(B)** Model prediction results.

**Figure 10** illustrates representative examples of the picking point localization results. As shown, the proposed algorithm demonstrates high accuracy and stability across a variety of complex backgrounds. It consistently maintains reliable localization performance under diverse environmental conditions. The algorithm effectively extracts the geometric structural features between the citrus fruit and its stem, enabling accurate inference of reasonable picking positions. These results highlight the method’s strong robustness and adaptability to varying environments. Experimental results indicate that the proposed approach exhibits promising application potential in real orchard settings, with a localization accuracy that meets the practical requirements for citrus harvesting operations.

**Figure 11:**
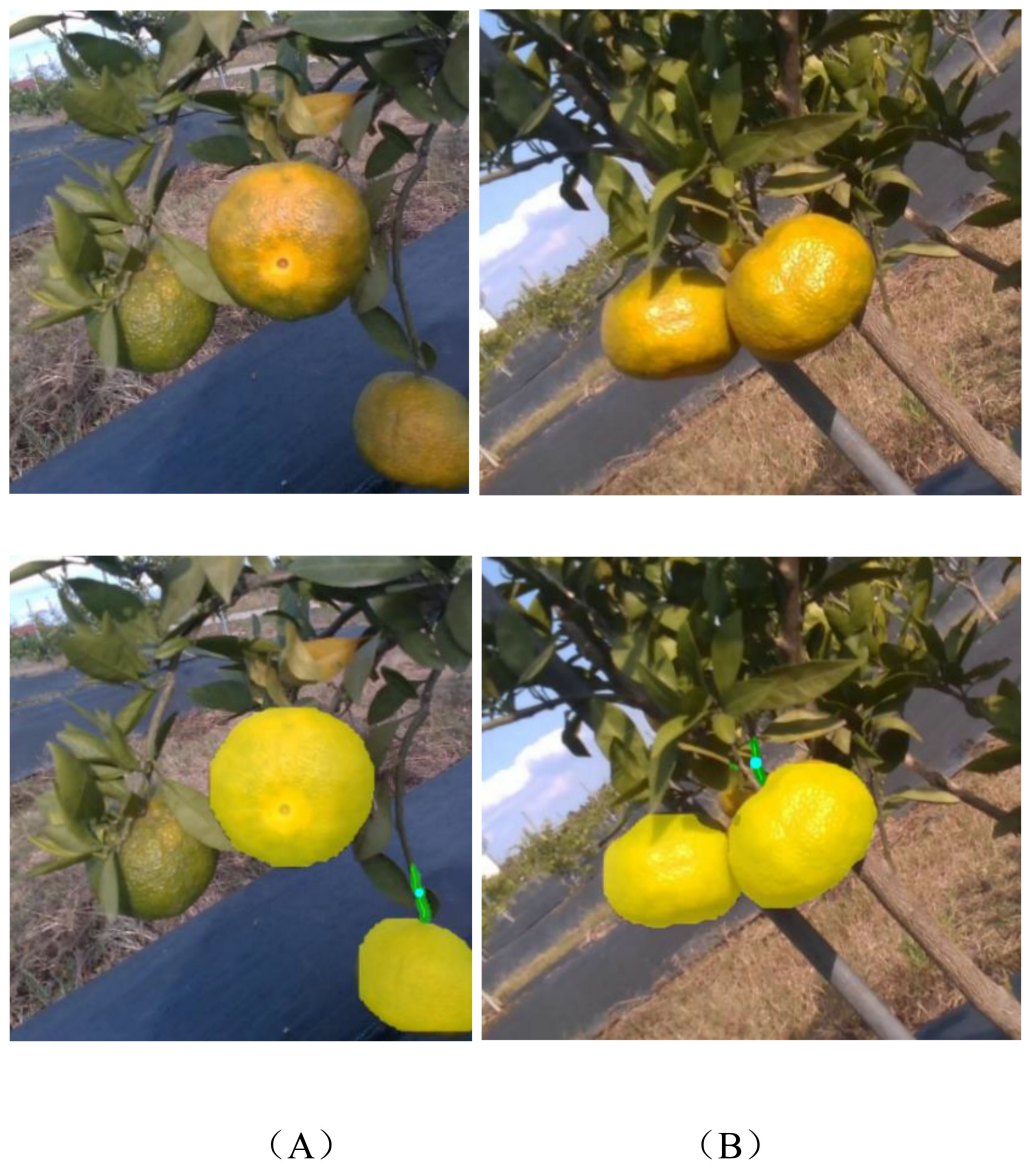
Examples of picking point localization failure in citrus. **(A)** Stem occlusion; **(B)** Stem segmentation error.

This study presents a visualization of typical failure cases in picking point localization, as shown in **Figure 11**. The main causes of localization failure are as follows: first, the stem is occluded by leaves or fruits. Although the fruit is successfully segmented, the connection between the fruit and its stem cannot be identified, resulting in failure to infer the picking point, as illustrated in **Figure 11a**. Second, inaccurate stem segmentation occurs; even if the stem is visible, segmentation errors affect the accuracy of picking point localization, as shown in **Figure 11b**. These observations indicate that occlusion and segmentation accuracy are key factors affecting robustness. Future research could incorporate multi-view image fusion or RGB-D information synergistic modeling to mitigate the impact of occlusion. Meanwhile, optimizing the segmentation algorithm by adopting more robust network architectures could enhance the model’s generalization ability in complex orchard environments.

## 4 Discussion

In recent years, with the rapid advancement of smart agriculture, automated fruit picking technology has gradually become a crucial component of orchard mechanization. However, in natural environments, citrus fruits and stems exhibit complex morphology and small dimensions, and are often subject to occlusion and background interference. These challenges result in limited accuracy and robustness for traditional object detection and segmentation methods, especially in stem recognition and precise picking point localization. Therefore, there is an urgent need for efficient and reliable solutions tailored to these issues.

This study focuses on the dwarf cultivar “Dafen No. 4” citrus and investigates segmentation methods for fruits and stems, as well as strategies for accurate picking point localization. A comparative analysis of various YOLO-based models was conducted on a citrus dataset, leading to the selection of an improved YOLOv8n-seg segmentation model for citrus recognition tasks. In related research, (Qi et al., 2024). enhanced the feature extraction capability and detection robustness of YOLOv8-seg by modifying the neck structure with BiFPN-based cross-layer connections and weighted fusion, and by replacing the SPPF module with a Soft-SPPF module. Similarly, (Si et al., 2024). improved stem segmentation performance under occlusion by incorporating a GCT module into the backbone and an EMA mechanism into the C2f module for enhanced multi-scale feature fusion. In this study, conventional convolution layers were replaced with GhostConv structures to reduce model parameters and size. Additionally, a CBAM and a small-object detection layer were introduced to improve the model’s sensitivity to critical regions and compensate for its limitations in fine-structure detection. Compared with other YOLO models (as shown in **Table 4**), the improved YOLOv8n-seg model achieved a mAP50 of 94.4%, effectively balancing detection accuracy and model efficiency. Despite these improvements, the model still faces challenges in complex scenarios such as severe occlusion, low lighting conditions, and overlapping fruits. Further work is required to enhance model robustness and address the generalization limitations of the current dataset.

To improve the accuracy of citrus picking point localization, this study builds upon the output of the improved YOLOv8n-seg model by incorporating a stem matching strategy based on geometric constraints. This method effectively reduces mismatches during stem recognition by utilizing the relatively stable spatial relationship between the fruit and its stem. Furthermore, the target stem region is structurally simplified, and geometric features are extracted to derive the stem’s central axis. The picking point is then determined based on this axis, providing a structurally stable and low-error localization result. Unlike traditional approaches that rely on the fruit center or rule-based estimation, the proposed method aligns more closely with practical harvesting requirements and accurately reflects the spatial characteristics of the stem. This localization strategy not only improves the precision of picking point determination but also provides a more reliable foundation for subsequent picking path planning and robotic arm control.

## 5 Conclusion

In this study, a citrus fruit and stem segmentation method based on an improved YOLOv8n-seg model was proposed. By integrating geometric constraints for stem matching, accurate localization of citrus picking points was achieved. The proposed method enhances localization accuracy while maintaining model lightweight characteristics, demonstrating strong robustness and practical application potential. The main conclusions are as follows:

1. The YOLOv8n-seg model was improved by replacing the original standard convolution layers with GhostConv modules to achieve a more lightweight network structure. In addition, the CBAM module and a small-object detection layer were introduced to enhance feature extraction for small targets. The improved model achieved precision, recall, mAP50, and F1-score of 96.56%, 90.91%, 94.43%, and 93.51%, respectively, for citrus fruits and stems. Compared with the original YOLOv8n-seg model, these metrics increased by 3.48%, 2.08%, 2.72%, and 2.70%, respectively. Among lightweight models including YOLOv5n-seg, YOLOv6n-seg, YOLOv9s-seg, and YOLOv8n-seg, the proposed model achieved the best segmentation performance.
2. Based on the segmentation results, a stem matching method guided by geometric constraints was proposed to achieve accurate localization of citrus picking points. This method establishes geometric relationships between fruits and stems to accurately match the target stem regions. The region of interest (ROI) corresponding to the matched stem is then subjected to morphological processing to extract the stem skeleton, and the midpoint of the skeleton is designated as the picking point. The approach was tested on 100 citrus images with a resolution of 1920 × 1080 collected by the camera. The results show that the method achieves an average picking point detection rate of 88.38%. With GPU support, the system completes segmentation and picking point localization in an average processing time of 373.25 milliseconds, demonstrating high real-time performance.

Overall, this study demonstrates promising progress in improving the accuracy of citrus segmentation and the efficiency of picking point localization. However, certain limitations remain in practical applications. During the citrus ripening process, various natural factors such as light conditions, nutrient distribution, and ventilation contribute to significant differences in fruit maturity even within the same orchard. These variations increase the complexity of determining the appropriate time for harvesting. Therefore, future research could focus on integrating citrus maturity detection with picking point decision-making algorithms. This approach would enable intelligent identification of mature fruits, thereby enhancing the operational performance and practical value of picking robots and supporting their large-scale deployment in real orchard environments.

## Data Availability

The original contributions presented in the study are included in the article/supplementary material. Further inquiries can be directed to the corresponding author.
